# Blockade of Fatty Acid Synthase Triggers Significant Apoptosis in Mantle Cell Lymphoma

**DOI:** 10.1371/journal.pone.0033738

**Published:** 2012-04-02

**Authors:** Pascal Gelebart, Zoulika Zak, Mona Anand, Andrew Belch, Raymond Lai

**Affiliations:** 1 Department of Laboratory Medicine and Pathology, University of Alberta and Cross Cancer Institute, Edmonton, Alberta, Canada; 2 Department of Oncology, University of Alberta and Cross Cancer Institute, Edmonton, Alberta, Canada; Wayne State University, United States of America

## Abstract

Fatty acid synthase (FASN), a key player in the *de novo* synthetic pathway of long-chain fatty acids, has been shown to contribute to the tumorigenesis in various types of solid tumors. We here report that FASN is highly and consistently expressed in mantle cell lymphoma (MCL), an aggressive form of B-cell lymphoid malignancy. Specifically, the expression of FASN was detectable in all four MCL cell lines and 15 tumors examined. In contrast, benign lymphoid tissues and peripheral blood mononuclear cells from normal donors were negative. Treatment of MCL cell lines with orlistat, a FASN inhibitor, resulted in significant apoptosis. Knockdown of FASN expression using siRNA, which also significantly decreased the growth of MCL cells, led to a dramatic decrease in the cyclin D1 level. β-catenin, which has been previously reported to be upregulated in a subset of MCL tumors, contributed to the high level of FASN in MCL cells, Interesting, siRNA knock-down of FASN in turn down-regulated β-catenin. In conclusion, our data supports the concept that FASN contributes to the pathogenesis of MCL, by collaborating with β-catenin. In view of its high and consistent expression in MCL, FASN inhibitors may hold promises for treating MCL.

## Introduction

Fatty acids play an important role in a variety of cellular processes. They serve as the building blocks for cell membranes, target anchor proteins to the cell membranes, function as precursors in the synthesis of lipid second messengers and act as important substrates for energy metabolism [1]. Fatty acids are also implicated in specialized biological functions including the production of lung surfactants and milk lipids [1]. There are two sources of fatty acids, namely the dietary source and that synthesized endogenously. The production of endogenous fatty acids is catalyzed by the multifunctional homodimeric lipogenic enzyme called fatty acid synthase (FASN) [2]. In this process, FASN catalyses the condensation of acetyl-CoA and malonyl-CoA to generate long-chain fatty acids, and the predominant product of FASN is palmitate, a 16-carbon fatty acid [1]. The *de novo* fatty acid synthesis is extremely active during embryogenesis and in proliferating fetal cells. In adult human tissues, FASN is mainly expressed in adipocytes, hepatocytes and hormone-sensitive cells such as lactating breast and cycling endometrial cells [3], [4]. In most of the other normal human tissues, FASN is expressed at a relatively low level, as these cells preferentially utilize dietary fatty acids [3], [4].

It has been recently found that FASN is highly expressed in many types of human solid tumors [5], [6], such as carcinomas of the breast [7], [8], prostate [9], colon [10], ovary [11], thyroid [12], lung [13] and stomach [10]. It has been suggested that a high level of FASN expression correlates with a shorter survival in patients with ovarian cancer [11]. These findings led to the hypothesis that the fatty acid synthetic pathway may contribute to tumorigenesis and FASN may be a useful anti-cancer target [5], [6], [9]. In support of this, an inhibitor of FASN and a FDA-approved anti-obesity drug, Orlistat, was reported to show antitumor activity [5]. Specifically, Orlistat has demonstrated potent anti-proliferative and pro-apoptotic effects in prostate, breast, colon, stomach and ovarian cancer cells, with no significant effects on normal cells [6]. Orlistat has also shown significant anti-tumor properties in a prostate cancer xenograft mouse model, without inducing signs of toxicity [14]. While the concept that FASN is a useful therapeutic target for epithelial cell malignancies is relatively supported, the role of FASN in hematologic cancer has not been extensively examined.

Mantle cell lymphoma (MCL) is a distinct type of B-cell non-Hodgkin's lymphoma defined by a constellation of pathologic, cytogenetic and clinical features [15]. One of the characteristic features of MCL is the recurrent chromosomal translocation, *t(11;14)(q13;q32)*, which brings the *cyclin D1* gene under the control of the enhancer of the immunoglobulin heavy chain gene, leading to over-expression of the cyclin D1 protein. While it is widely accepted that cyclin D1 plays an important role in the pathogenesis of MCL, accumulating evidence suggests that MCL often has defects in many other cellular processes, such as those involved in cell-cycle regulation, apoptosis and DNA repair [16], [17]. With regard to apoptosis, MCL is well known to be resistant to apoptosis induced by a variety of conventional chemotherapeutic agents [17]. Recent studies have revealed a number of biochemical defects that may contribute to its relatively high resistance to apoptosis [18], including constitutive activation of the NFκB pathway [19]–[21], overexpression of several anti-apoptotic proteins and the absence of Fas receptor [22]. Aberrant cellular signaling such as the PI3K/Akt pathway also may contribute to the chemo-resistance of MCL [23], [24]. Despite the advent of several new therapeutic agents [25], a significant proportion of MCL patients continues to have a relatively poor clinical outcome [17]. Thus, there is a need to continue to develop new therapeutic strategies for this disease. In this study, we found that FASN is highly and consistently expressed in MCL cell lines and tumors. Importantly, blockade of FASN can induce significant apoptosis in MCL. Our findings suggest that FASN may represent a useful therapeutic target for MCL.

## Materials and Methods

### 1. Cells, tissue culture and FASN inhibitors

Previously described MCL cell lines, including Jeko-1, Mino, SP53 and Rec-1, were used in this study [26]. Briefly, these cell lines are positive for cyclin D1, and they carry an mature B-cell immunophenotype and the *t(11;14)(q13;q32)* cytogenetic abnormality. All of these cell lines are negative for the Epstein-Barr virus nuclear antigen and they were grown in RPMI 1640 supplemented with 10% fetal bovine serum (FBS) and glutamine. To test the sensitivity of MCL cells to FASN inhibitors, MCL cells were plated at a density of 1×10^5^ cells/mL and treated with control diluent (DMSO) or different concentrations of two FASN inhibitors, namely C75 (Cayman Chemical Company, Ann Arbor, MI) and Orlistat (Alexis Biochemicals, San Diego, CA). CellTiter-Blue fluorescence was used to monitor the cell viability according to the manufacturer's protocols (Promega, Madison, WI).

### 2. MCL patient samples and immunohistochemistry

All MCL primary tumors samples were diagnosed at the Cross Cancer Institute and the diagnostic criteria were based on those described in the World Health Organization Classification Scheme [27]. Formalin-fixed, paraffin-embedded tumor samples from 15 cases of MCL were subjected to immunohistochemistry based on a protocol previously published [28]. Briefly, tissue sections of 3–4 µM thickness were deparaffinized and hydrated. Heat-induced epitope retrieval was obtained by using a pressure cooker and citrate buffer (pH 6.0). The endogenous peroxidase activity was blocked using 0.3% H_2_O_2_ in methanol for 5 minutes. Tissue sections were then incubated with a mouse monoclonal anti-FASN antibody (Santa-Cruz Biotechnology, Santa Cruz, CA, 1∶200) overnight at 4°C in a humidified chamber. After 2 washes with phosphate buffered saline (PBS, pH 7.5), tissue sections were incubated with biotinylated linked universal secondary antibody and subsequently with streptavidin–HRP complex as per the manufacturer's instructions (LSAB+ system, Dako, Burlington, Ontario, Canada). Tissue sections were incubated with 3,3′-diaminobenzidine/H_2_O_2_ (Dako) for color development and counter-stained with hematoxylin. To assess the sensitivity of leukemic MCL to a FASN inhibitor, peripheral blood mononuclear cells (PBMC) from 3 leukemic patients (all of whom had an absolute lymphocyte count of >5×10^9^/L) were harvested using Ficoll-Paque density centrifugation, plated at a density of 1×10^5^ cells/mL in culture medium, and treated with either DMSO or different concentrations of Orlistat.

### 3. Apoptotic assays

The FITC-Annexin V Apoptosis detection kit (BD Biosciences Pharmingen, San Diego, CA) was used to detect the phosphatidylserine translocation from the inner to the outer leaflet of the plasma membrane. Briefly, 1×10^6^ cells were diluted in 100 µL of Annexin V buffer, to which 5 µL of Annexin V-FITC was subsequently added. After incubation for 15 min at room temperature in the dark, 400 µL of additional binding buffer was added. Flow cytometry analysis was conducted within 1 hour. Caspase 3/7 activities were measured using the Apo-ONE Homogeneous Caspase-3/7 Assay kit (Promega) according to the manufacturer's protocol. Briefly, 100 µL of Caspase 3/7 Apo-One reagent was added to 100 µL of cells culture treated with or without FASN inhibitors. After incubation, the fluorescence of each sample was measured in a fluorescent plate-reading FLUOstar Optima (BMG Labtechnologies, Offenburg, Germany). Triplicate experiments were performed and results are presented as the mean ± the standard deviation.

### 4. siRNA and transfection

Cells were transfected with a pool of 4 siRNA targeting FASN or β-catenin (Dharmacon Inc., Lafayette, CO). Cells treated with scrambled siRNA served as the negative control. Transient transfections of MCL cells (5×10^6^ cells) were performed using the Electro square electroporator, BTX ECM 800 (225V, 8.5 ms, 3 pulses, Harvard apparatus). A concentration of 200 pmol of siRNA per 1×10^6^ cells was used. The efficiency of target gene inhibition was assessed using Western blot analysis.

### 5. Western blot analysis

Cells harvested for Western blot analysis were washed with ice-cold PBS and lysed in buffer containing 1% Triton X-100 and a complete protease and phosphatase inhibitor cocktail. Protein samples were electrophoresed through 10% SDS-polyacrylamide gels and transferred to nitrocellulose. The membrane was stained with 0.05% Ponceau S (Sigma-Aldrich, Oakville, Ontario, Canada) to ensure equal protein loading. Primary antibodies reactive with the following antigens were used: cyclin D1, poly(ADP-ribose) polymerase-cleaved (PARP), cleaved caspase 3, cleaved caspase 7 (Cell Signaling Technology, Beverly, MA), PPARα (Rockland Immunochemicals, Gilbertsville, PA) and β-actin, (Santa Cruz Biotechnology). Immunoreactivity was detected using peroxidase-conjugated anti-mouse or anti-rabbit IgG and visualized by enhanced chemiluminescence (Pierce, Rockford, IL).

### 6. Reverse transcriptase-polymerase chain reaction (RT-PCR) and Real-time reverse transcription-PCR

RT-PCR was used to detect *FASN* mRNA in MCL cell lines. Total RNA was prepared with the Trizol (Invitrogen, Burlington, Ontario, Canada) in accordance with the manufacturer's suggested protocol. Briefly, cDNA synthesis was carried out for 50 minutes at 42°C using the superscript reverse transcriptase II (Invitrogen). The PCR was performed for 30 cycles in a thermal cycler (Applied Biosystems, Streetville, On, Canada), with each consisting of denaturation (94°C for 1 min), primer annealing (60°C for 1 min) and DNA extension (72°C for 1 min). Amplified products were electrophoresed in 2% agarose gel containing ethidium bromide and visualized using Alpha Imager 3400 (Alpha Innotech, San Leandro, CA). The following sets of primers were used: *FASN*- forward: 5′-AAGGTCATCCCTGAGCTGAA-3′; *FASN* reverse: 5′-CCCTGTTGCTGTAGCCAAAT-3′ (expected size, 292 bp); *Glyceraldehyde-3-phosphate dehydrogenase* (*GAPDH*) was used as internal control, *GAPDH* housekeeping primers forward: 5′-AAGGTCATCCCTGAGCTGAA-3′, reverse: 5′-CCCTGTTGCTGTAGCCAAAT-3′ (expected size, 316 bp). Twenty-four hours after transfection of β-catenin siRNA, total RNA isolated from cells was reverse transcribed as described above. The cDNA was then amplified for: *β-catenin* primers forward: 5′-CCCACTAATGTCCAGCGTTT-3′, reverse: 5′-CTCACGATGATGGGAAAGGT-3′.

### 7. Flow cytometry

MCL cells were fixed in the CytoFix Buffer from Becton Dickinson Biosciences (Franklin Lakes, NJ) washed in cold PBS, centrifuged, and re-suspended in the fluorescence-activated cell scanner (FACS) staining buffer purchased from Becton Dickinson. Cells were incubated with primary antibodies for 60 minutes at 4°C in the dark, and washed twice using cold buffer between incubations. The following antibodies were employed: unconjugated mouse IgG1 as the isotype control (10 µg/mL, Santa Cruz biotechnology), unconjugated mouse anti-human CD19 (Becton Dickinson Biosciences) and anti-human FASN (R and D systems, Minneapolis, MN). Flow cytometry was performed using the FACScan (Becton Dickinson Biosciences) and the data was analyzed using the accompanying CELLQuest software as per manufacturer's guidelines.

### 8. Statistical analysis

Statistical analysis was performed using the StatView 4.5 software. Results are expressed as mean ± standard deviations. Student's *t*-test is used to assess the statistical significance whenever appropriate. Differences between two sets of experimental data are considered to be statistically significant when the *P* value is less than 0.05. The regression coefficient (R) is determined by linear regression analysis.

## Results

### 1. FASN is highly expressed in MCL but not normal lymphocytes

**Figure 1:**
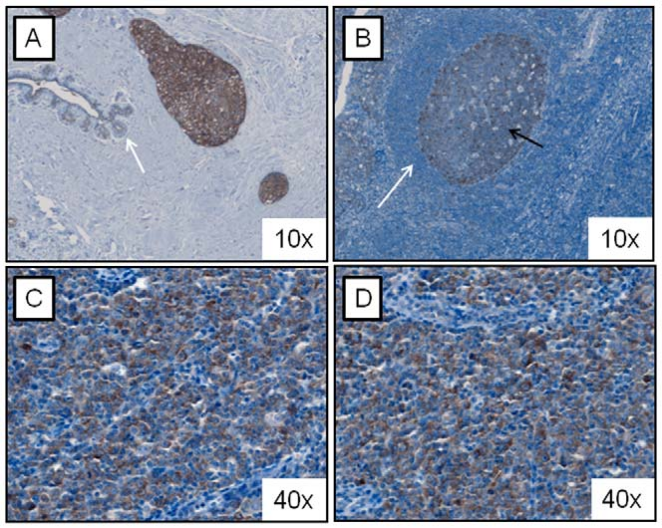
FASN was highly expressed in MCL patient samples. A) Immunohistochemical studies revealed strong cytoplasmic FASN staining in breast carcinoma cells (i.e. positive control). In comparison, the adjacent benign mammary epithelial cells showed only faint cytoplasmic staining (white arrow). B) Normal tonsillar lymphoid tissue showed no definitive staining in the mantle zones (white arrow). The germinal center cells were weakly positive (black arrow). C and D) Two MCL tumors showed relatively homogeneous FASN cytoplasmic staining.

Using immunohistochemistry, the expression of FASN was examined in 15 MCL tumors, and the results are illustrated in figure 1. Breast carcinomas were used as the positive controls. We found that all 15 cases of MCL showed intense cytoplasmic staining throughout the tumors. In contrast, with the exception of the germinal centers, lymphocytes in benign tonsillar tissues (n = 5), including those in the mantle zone, were negative.

**Figure 2:**
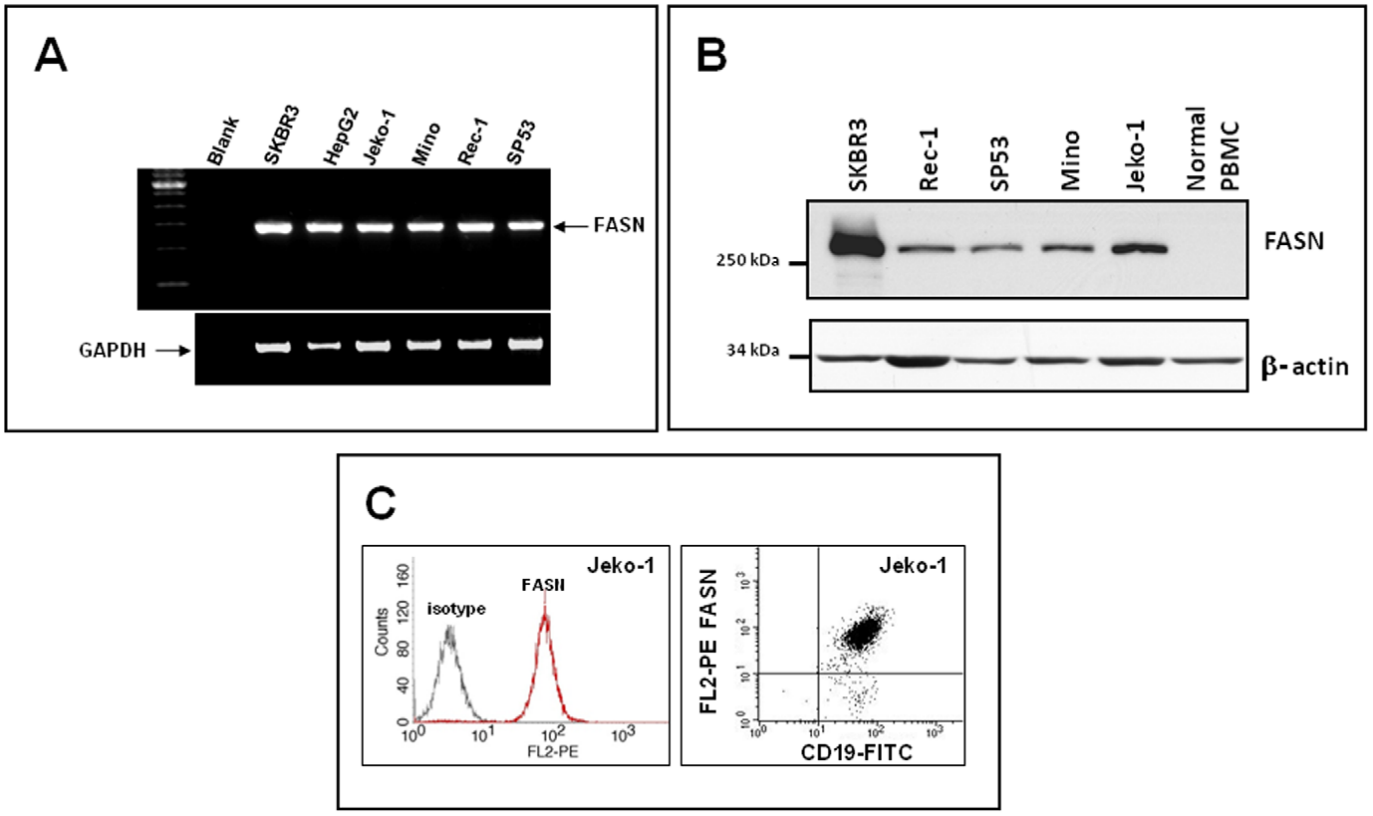
FASN was highly expressed in MCL cell lines. A) RT-PCR studies demonstrated the high level of *FASN* mRNA in four MCL cell lines. SKBR3 (a breast cancer cell line) and HepG2 (a hepatocarcinoma cancer cell line) were used as positive controls. B) The FASN protein expressed in MCL cell lines was readily detectable by Western blots. SKBR3 was used as a positive control. In contrast, protein lysates prepared from isolated peripheral blood mononuclear cells (PBMCs) from a healthy donor were negative for the FASN protein. C) Flow cytometry analysis revealed the expression of FASN in Jeko-1 cells.

We then analyzed FASN expression in MCL cell lines using RT-PCR. As shown in figure 2A, the *FASN* transcript was readily detectable in SKBR3 and HepG2 (a breast cancer cell line and a hepatocarcinoma cell line, respectively), which served as the positive controls. The *FASN* transcript was readily detectable in all 4 MCL cell lines examined. By Western blots, FASN protein was detectable in all 4 MCL cell lines whereas PBMC from a healthy donor were negative (figure 2B). By flow cytometry, we were able to detect a relatively high level of FASN expression in Jeko-1 cells; in contrast, CD19-positive B-cells in the peripheral blood from a healthy donor were negative for FASN (not shown).

### 2. Pharmacologic inhibition of FASN induces significant inhibition in cell-growth in MCL cell lines

**Figure 3:**
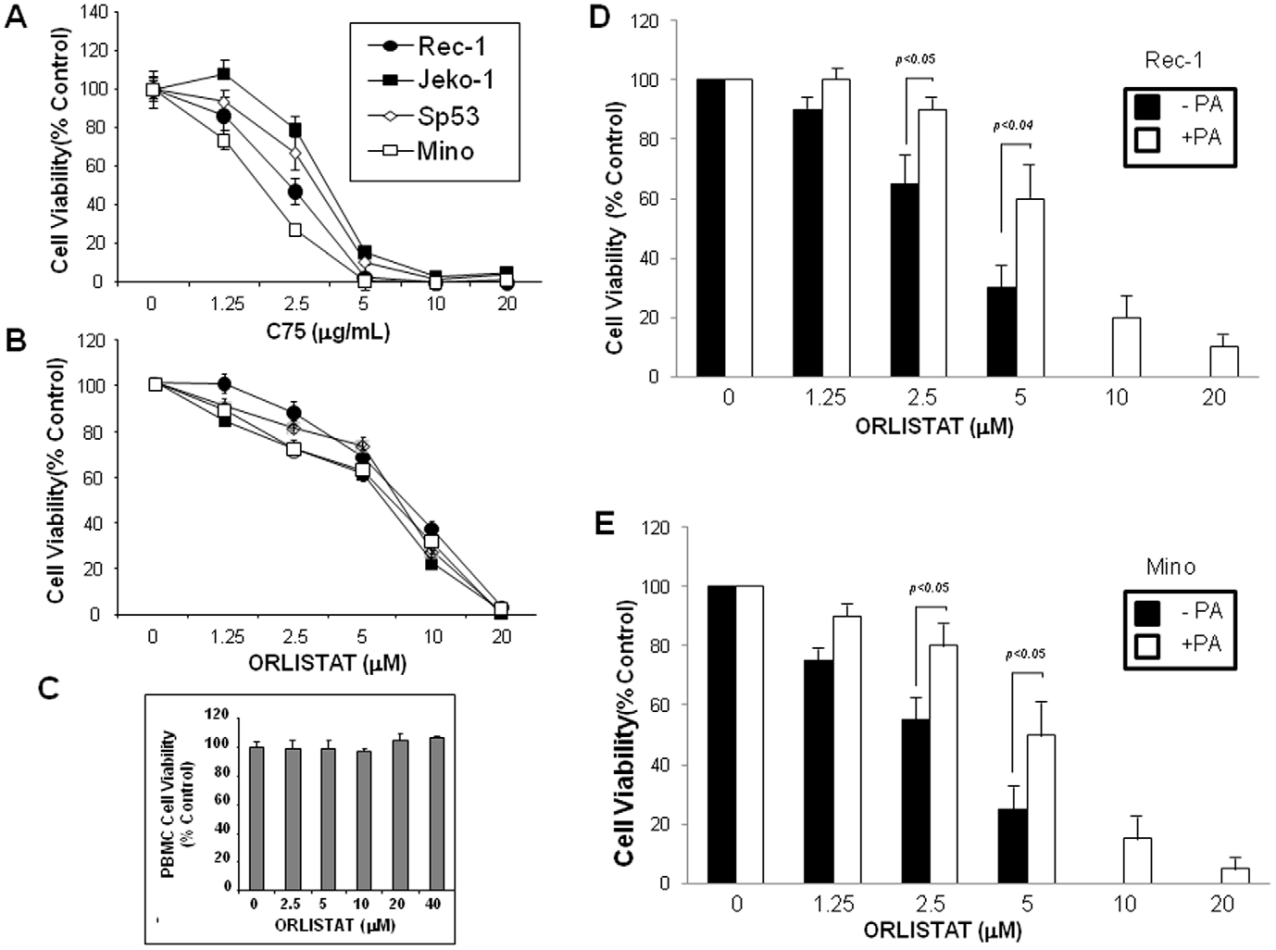
FASN inhibitors induced cell death of MCL cells. A and B) Four MCL cell lines were treated with two widely-used FASN inhibitors, namely C75 and Orlistat, in concentrations ranging to 0 to 20 µg/mL for C75 and 0 to 20 µM for Orlistat. At 48 hours, the number of viable cells, as determined by using the trypan blue exclusion assay, decreased in a dose-dependent manner. Experiments were performed in triplicate and the means +/− standard deviations are shown. C) In contrast to MCL cells, PBMC showed no appreciable effects to Orlistat. D and E) To establish a more direct link between fatty acid production and the observed loss of cell viability, we pre-treated the cells with 30 µM of palmitic acid (labeled as PA). Palmitic acid significantly blocked the cell-growth inhibitory effects of Orlistat, but it did not completely abrogate the effects of this drug in MCL cells.

To determine the biological importance of FASN in MCL cells, we inhibited FASN in MCL cell lines using two previously published pharmacological inhibitors, namely C75 and Orlistat. We first determined the concentrations of these agents at which the viability of the cells can be reduced to 50% (i.e. IC50). As shown in figure 3, C75 and Orlistat induced a significant reduction in the number of viable cells in a dose-dependent manner. At 24 hours after the treatment, the IC50 value was in the range of 2.5–5 µM for C75 and 5–10 µM for Orlistat. As illustrated in figure 3C, Orlistat induced no detectable loss of cell viability in PBMC from a healthy donor. To establish a more direct link between fatty acid production and the observed loss of cell viability, we pre-treated the cells with 30 µm of palmitic acid. As shown in figure 3D and 3E, this pre-treatment significantly reduced the cell-growth inhibitory effects of Orlistat. The observation that palmitic acid did not completely abrogate the effects of Orlistat suggests that Orlistat probably inhibited MCL cells via mechanisms unrelated to fatty acid production.

**Figure 4:**
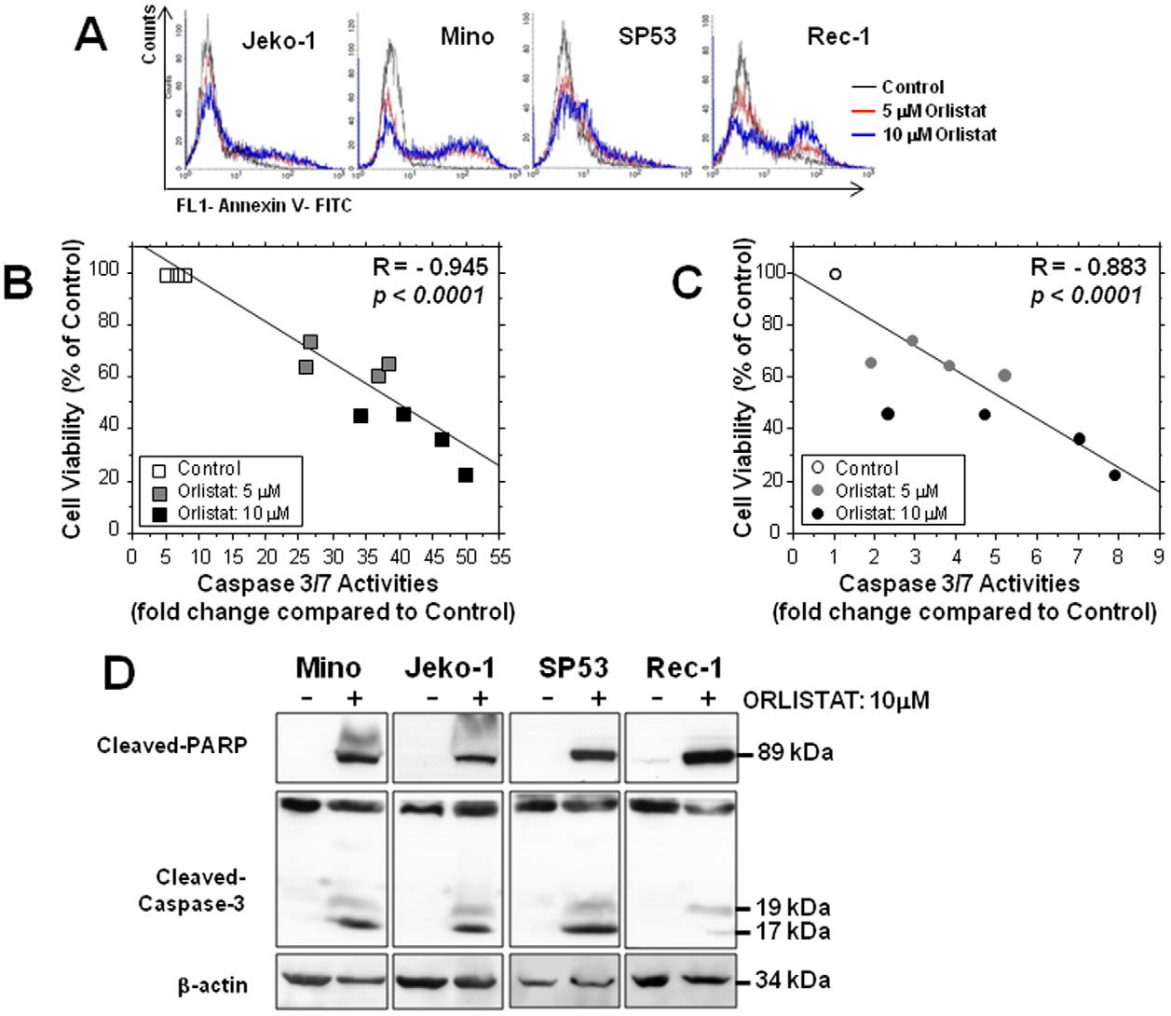
FASN inhibitors induced apoptosis of MCL cells. The four MCL cell lines were treated with two different concentrations of Orlistat. A) The degree of apoptosis was assessed based on the cell surface expression of Annexin V in MCL cell lines detectable by flow cytometry. In addition, the cell viability in two MCL cells, Rec-1(B) and Mino (C) treated with 5 µM or 10 µM Orlistat for 48 hours, was significantly correlated with the enzymatic activity of caspase 3/7. D) Using western blot, cleaved capase 3 and PARP were detectable in MCL cells treated with 10 µM Orlistat.

We then assessed if the FASN inhibitors induce apoptosis in MCL cells. As shown in figure 4A, MCL cells treated with 5 or 10 µM of Orlistat for 24 hours contained a subset of Annexin V-positive cells. Furthermore, we found that increasing concentrations of Orlistat induced a dose-dependent increase in Annexin V-positive cells, with a corresponding decrease in the cell viability (p<0.0001; figure 4A). We also employed an *in-vitro* enzymatic caspases 3/7 assay, which provides a quantitative assessment of apoptotic activity. Following 24 hours incubation with 5 or 10 µM of Orlistat, the caspase 3/7 activities in two MCL cell lines (Rec-1 and Mino) were increased by up to 50 and 8 fold, respectively, with a corresponding decrease in cell viability (p<0.0001; figure 4B and 4C). By Western blots, we found that treatment with 10 µM of Orlistat induced cleavage of caspase-3 and PARP in all four MCL cell lines (figure 4D).

### 3. Knockdown of FASN by siRNA induces cell death of MCL and cyclin D1 downregulation

**Figure 5:**
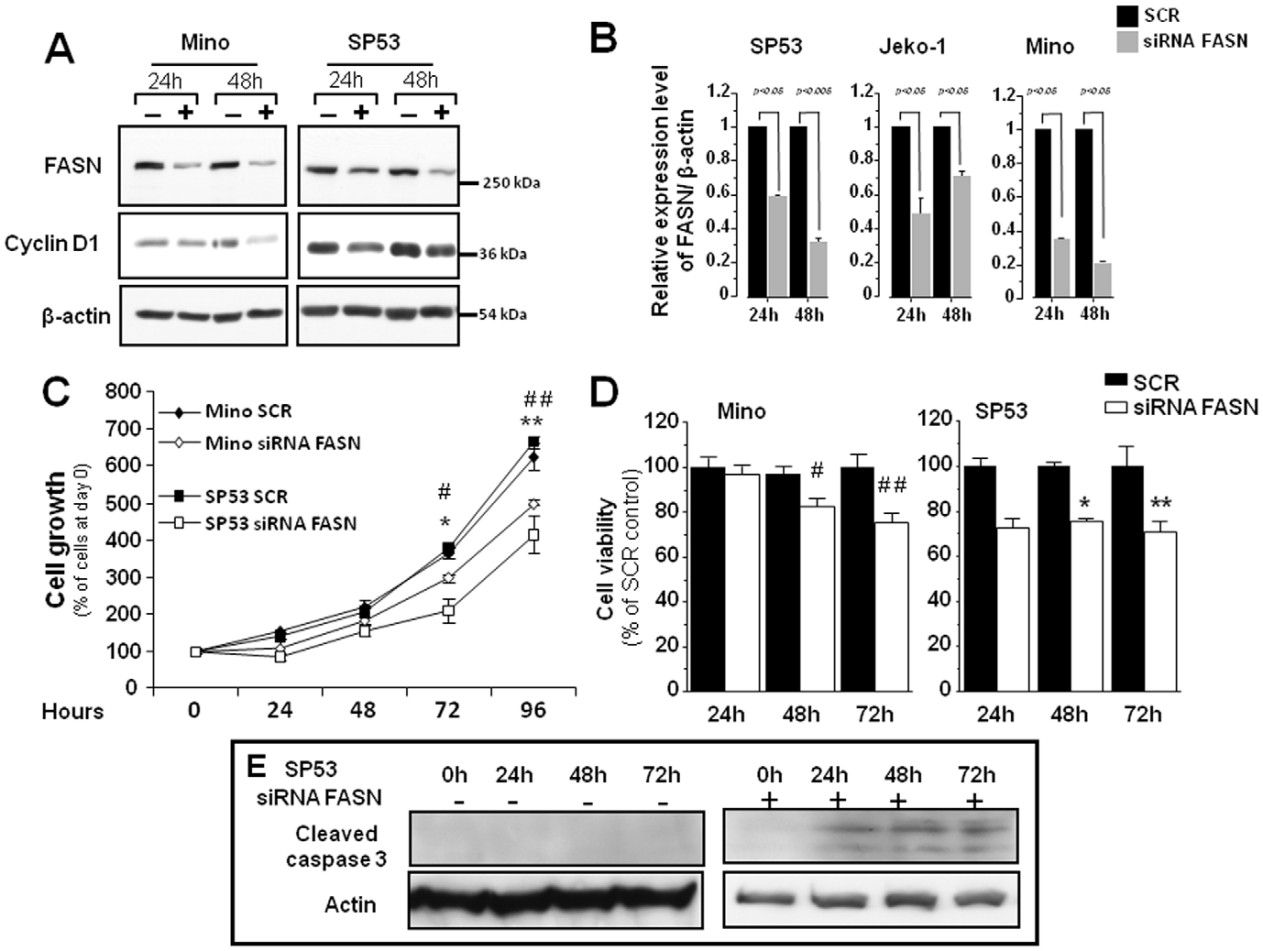
Knockdown of FASN decreased the expression of cyclin D1 and induced cell death of MCL cells. A) Two MCL cell lines, Mino and SP53, were treated with siRNA to knockdown FASN. The expression of FASN and cyclin D1 were assessed by western blot. The expression of FASN and cyclin D1 was dramatically decreased after *FASN* siRNA treatment. B) Quantification studies showed that FASN expression was decreased in MCL after treatment with *FASN* siRNA as compared to cells treated with scramble siRNA. Triplicate experiments were performed. (C) Two MCL cell lines were treated with *FASN* siRNA for up to 96 hours. The cell growth was evaluated using trypan blue exclusion assay. Knockdown of FASN expression significantly decreased the growth of MCL cells (for Mino cells - ^#^ p<0.005 at 72 hours; ^##^ p<0.005 at 96 hours; for SP53 cells - * p<0.05 at 72 hours; ** p<0.05 at 96 hours). D) Treatment of two MCL cell lines with *FASN* siRNA significantly decreased the number of viable cells, as assessed by using the MTS assay. The differences are statistically significant for Mino (^#^ p<0.05 at 48 hours and ^##^ p<0.005 at 72 hours) and for SP53 (* p<0.05 at 48 hours and ** p<0.005 at 72 hours). Triplicate experiments were performed. E) Treatment of SP53 cell line with siRNA *FASN* induced caspase 3 cleavage.

To further determine the biological significance of FASN in MCL, we blocked FASN expression using siRNA and assessed the biological effects. By Western blots, siRNA treatment induced a substantial reduction in FASN protein expression, ranging from 30–60% within 24 hours of transfection (figure 5A and 5B). As shown in figure 5C, blockade of FASN expression using siRNA significantly decreased cell growth, at approximately 20% for Mino and 40% for SP53 cells at 96 hours post-transfection (figure 5C). Simultaneously, the number of viable cells, determined by trypan blue staining, was reduced significantly after treatment with specific *FASN* siRNA (*P*<0.05) in Mino and SP53 cells as compared with cells transfected with scramble siRNA (figure 5D). As shown in figure 5E, inhibition of FASN expression by siRNA in SP53 cells induced cleavage of caspase-3.

### 4. Orlistat induces significant apoptosis in leukemic MCL cells

**Figure 6:**
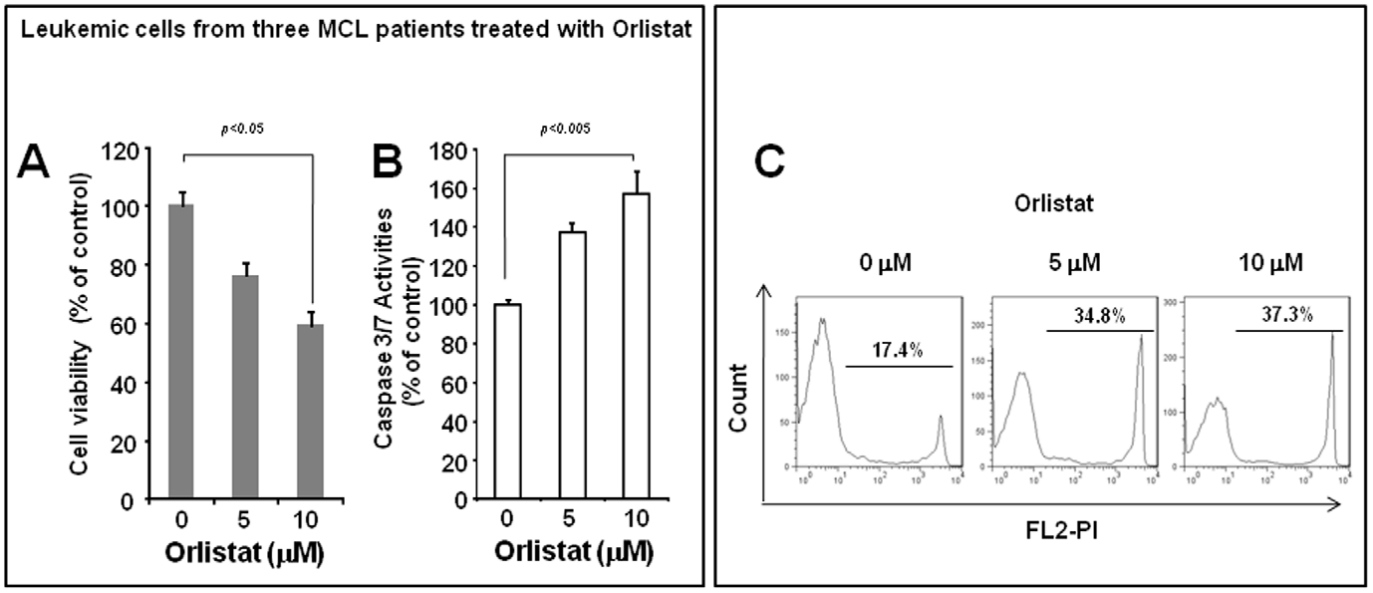
Orlistat induced cell death in primary leukemic MC cells. A) Leukemic cells from three patients were treated with two different concentrations of Orlistat. The number of viable cells was assessed by trypan blue assay after 24 hours of treatment. Results illustrated represented the ‘pooled’ data of these three samples. B) In the same experiment, the caspase-3/7 activity was assessed using a commercially available Caspase-3/7 Apo-One kit. Again, results illustrated represented the ‘pooled’ results of three samples. Triplicate experiments were performed. C) Representative results of propidium iodine staining of leukemic MCL samples treated with Orlistat showed a dose-dependent increase of staining detectable by flow cytometry.

To investigate the effect of FASN inhibition on primary MCL cells, leukemic MCL cells from three patients were treated with different concentrations of Orlistat, and the results are illustrated in figure 6. Orlistat induced a significant decrease in the cell viability, as determined by trypan blue staining (figure 6A). This also correlated with increased caspase-3/7 activity (figure 6B). In one of these three patients, we also performed propidium iodide (PI) staining (figure 6C), and increasing concentrations of Orlistat induced a higher proportions of PI-positive cells.

### 5. FASN inhibition decreases β-catenin expression

**Figure 7:**
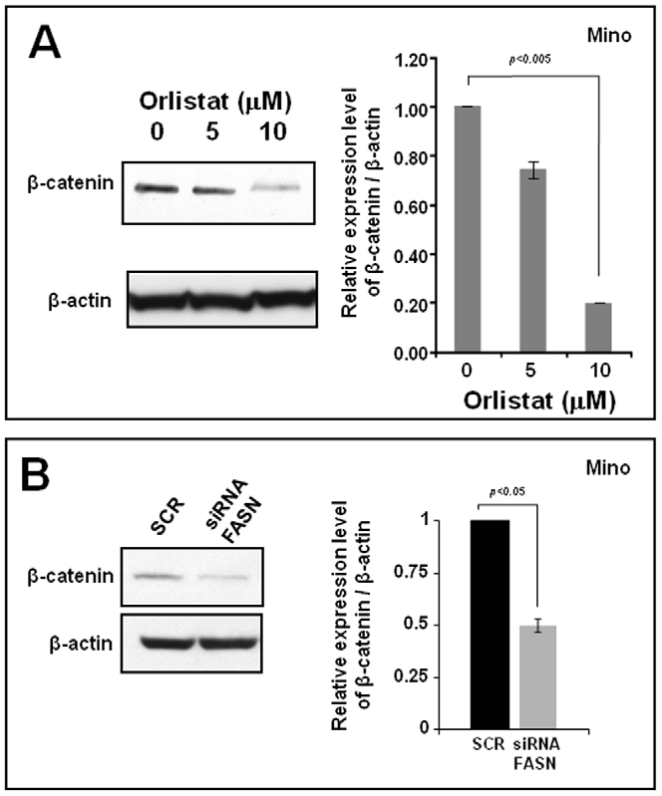
FASN inhibition decreased β-catenin expression. The MCL cell line Mino was treated with Orlistat (A) or siRNA (B) to inhibit or knock-down FASN, respectively. The β-catenin protein expression was determined by western blot after 48 hours of treatment. FASN inhibition by either Orlistat or siRNA substantially decreased the protein expression of β-catenin. Triplicate experiments were performed.

It has been recently observed that FASN is associated with β-catenin stabilization in prostate cancer [29]. In addition, we have recently reported that β-catenin is activated in a subset of MCL [28]. Thus, we hypothesize that the relatively high level of FASN expression in MCL cells may be linked to β-catenin in these cells. To test this possibility, Mino, SP53 and Jeko-1 cells were transfected with *FASN* siRNA or scramble siRNA. As shown in figure 7A, inhibition of FASN using Orlistat induced a dose-dependent decrease in the protein level of β-catenin (figure 7A). Similar results were observed when the cells were transfected with the siRNA to knock-down FASN (figure 7B).

### 6. β-catenin contributes to FASN expression in MCL

**Figure 8:**
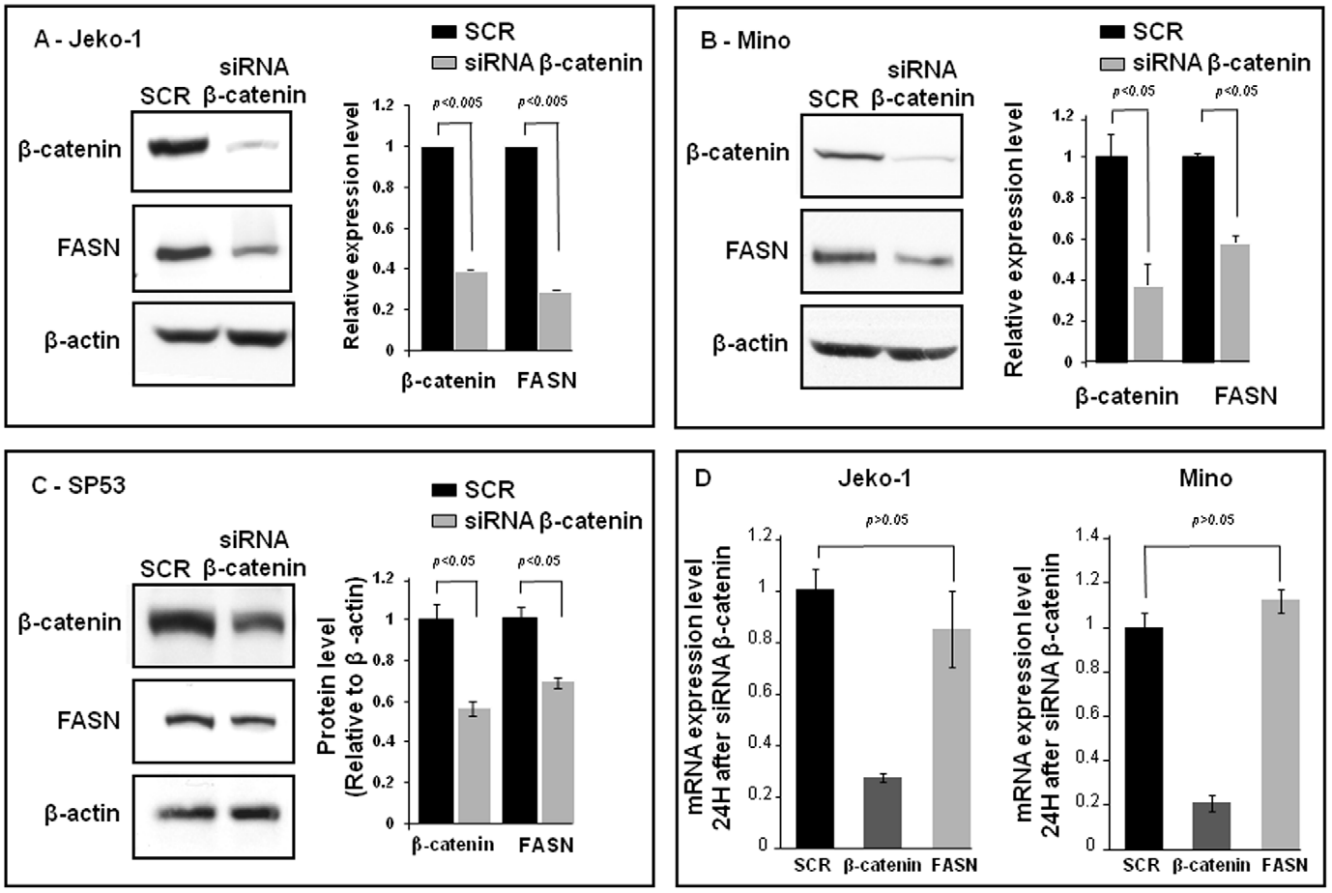
Knock-down of β-catenin substantially decreased FASN protein expression in MCL cells. A–C) Three MCL cell lines were treated with siRNA β-catenin or scramble siRNA and the expression of FASN was evaluated by Western blot. siRNA treatment induced a substantial decrease in FASN protein in all the three MCL cell lines studied. Triplicate experiments were performed. D) The mRNA level of *FASN* was determined in MCL cell lines after treatment with siRNA β-catenin. Knock-down of β-catenin did not significantly decrease the mRNA level of *FASN*. Triplicate experiments were performed.

**Figure 9:**
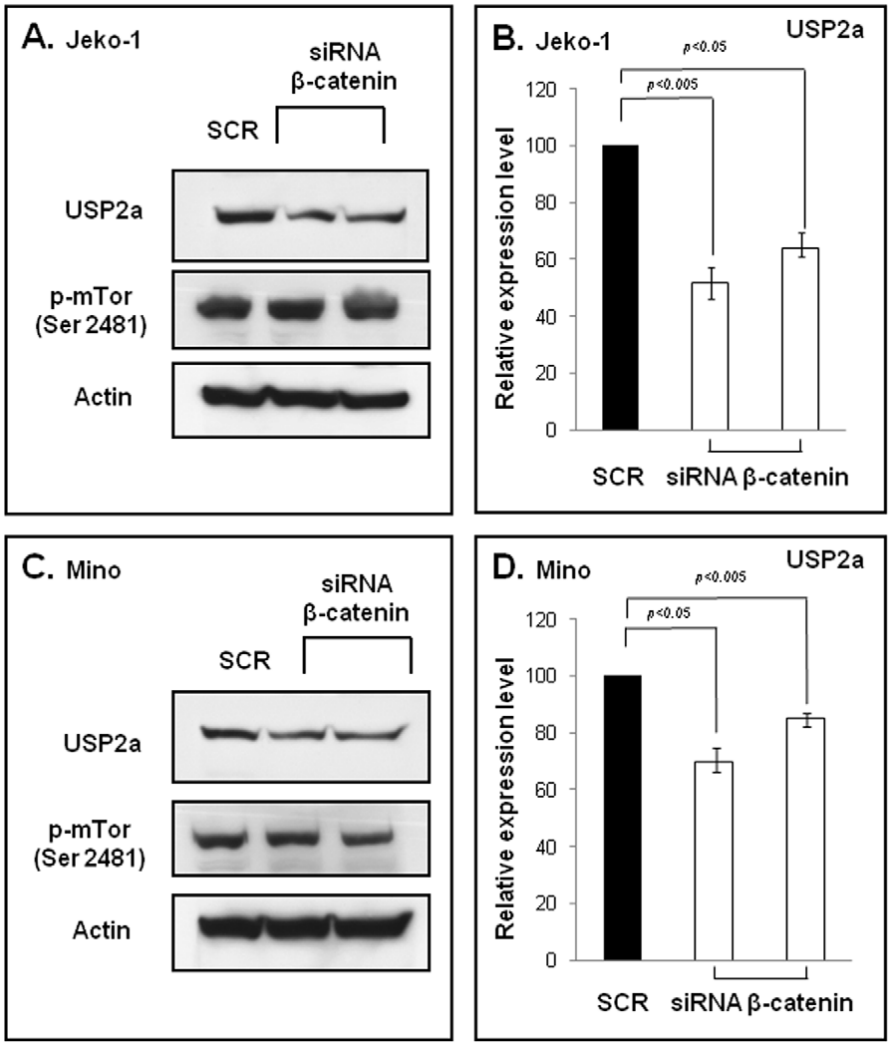
β-catenin decreased USP2a protein expression in MCL cells. A and B) two MCL cell lines were treated with two different siRNA β-catenin species or scramble siRNA, and the expression of phospho-mTor (ser2481) and USP2a were evaluated by Western blots. siRNA treatment induced a substantial decrease in the levels of the USP2a protein in both cell lines. In contrast, no appreciable modulation of phospho-mTor (as a surrogate marker of mTor activation) was observed. Triplicate experiments were performed and represented results are illustrated.

Our analysis of the promoter region of the *FASN* gene has revealed the presence of TCF/LEF binding sites. Thus, we hypothesize that β-catenin may in turn contribute to the overexpression of FASN. To test this possibility, MCL cell lines were treated with β-catenin siRNA or scramble siRNA. As shown on figure 8, inhibition of β-catenin expression using siRNA led to a substantial decrease of FASN protein expression. Similar results were observed when two individual β-catenin siRNA were used (figure S1). As shown in figure 8D, β-catenin siRNA did not induce appreciable downregulation of *FASN* mRNA, suggesting β-catenin regulates the FASN protein level primarily via post-transcriptional mechanisms. Since previous reports have shown that FASN can be regulated by the mTor signaling pathway and USP2a-induced protein stabilization in cancer cells [30], [31], we asked if the effect on FASN expression mediated by β-catenin involves these two pathways. As shown in figure 9, siRNA knock-down of β-catenin expression led to a substantial down-regulation of USP2a protein expression, and this finding suggests that β-catenin may indeed increase the protein expression of FASN by inhibiting its protein degradation. In contrast, the same treatment did not result in detectable alterations of mTor activation in MCL cells.

## Discussion

FASN, a protein playing important roles in the endogenous fatty acid production, has been recognized to contribute to oncogenesis in solid tumors. It has been well documented that FASN is over-expressed in many types of epithelial malignancies, such as those derived from the prostate and breast. However, the relevance of FASN in hematologic malignancies has not been fully examined. The expression status and functional significance of FASN has never been examined in MCL. In the present study, we report that FASN is consistently expressed at a high level in MCL cell lines and tumors. These findings are in contrast with the undetectable FASN level in benign lymphoid tissues, including normal lymphocytes present in the mantle zone. The lack of FASN expression in normal peripheral blood mononuclear cells also has been previously reported [32]. Of note, the high expression level of FASN is not a universal phenomenon in hematologic malignancies. Based on our literature search, we identified reports describing a high level of FASN expression in myelomas [32] and in diffuse large B-cell lymphomas [33]. In contrast, the expression of FASN was not detected in cDNA microarray studies of chronic lymphocyte leukemia, another CD5-positive B-cell lymphoproliferative disorder [34].

We found that the high level of FASN expression in MCL is biologically important. Specifically, our results suggest that FASN confers anti-apoptotic effects in these cells. In support of this concept, the use of FASN pharmacologic inhibitors (including C75 and Orlistat) was found to induce apoptosis in a dose-dependent fashion in all MCL cell lines examined as well as 3 samples of primary MCL leukemic cells. FASN inhibition using siRNA was also found to induce apoptosis, associated with a downregulation of cyclin D1 and β-catenin. We noted that the siRNA-induced inhibition of FASN did not induce apoptosis as efficient as the two pharmacological drugs did. This observation can be explained by the fact that *FASN* siRNA did not completely abrogate the expression of FASN; approximately 20 to 30% of FASN protein remained to be detectable in MCL cell lines after the siRNA treatment. Alternatively, it is likely that the pharmacologic agents had off-target effects, as this concept is supported by our observation that pre-treatment of palmitic acid did not completely abrogate the cell-growth inhibitory effects of Orlistat (figure 3C and 3D).

The mechanisms by which FASN is upregulated in cancer cells have been described in a number of epithelial malignancies. For instance, the Akt signaling pathway and HER-2 have been reported to play a role in upregulating FASN expression in breast cancer [35] as well as in thyroid papillary carcinoma [12]. While the Akt pathway may contribute to the high expression level of FASN in MCL cells, we believe that it is highly unlikely that it is the sole mechanism, as constitutive activation of the Akt pathway is largely restricted to blastic MCL cases in one study [36]. To further examine the mechanism by which FASN expression is driven in MCL cells, we analyzed the promoter region of the *FASN* gene, in an attempt to identify specific transcriptional factors that may be responsible for the high level of FASN expression in MCL cells. Our analysis led us to identify the consensus binding sequence for β-catenin (unpublished finding). In keeping with the importance of β-catenin in inducing FASN in MCL, we found that inhibition of β-catenin in MCL cells using siRNA led to a substantial decrease in the protein level of FASN (Figure 8 and figure S1). Rather surprisingly, the level of *FASN* mRNA was not significantly downregulated. These observations suggest that β-catenin does not exert transcriptional control over *FASN* expression in MCL cells; instead, it is likely that it regulates the FASN protein level by modulating its stability/protein degradation. In this regard, two previous reports have observed that the protein expression of FASN in prostate and breast cancer is regulated at the post-transcriptional level [30], [31]. One of these two reports highlights the role of USP2a (ubiquitin-specific-protase 2a), an isopeptidase [30], [31]. In light of this information, we asked if β-catenin may contribute to the overexpression of FASN protein by decreasing USP2a. As shown in figure 9a, it turns out to be the case. Interestingly, our analysis of the human promoter of *USP2a* has revealed the presence of TCF/LEF binding site (unpublished finding). Taken together, it appears that β-catenin increases the protein expression of FASN in MCL cells by promoting it stabilization via modulation of USP2a. We also asked if the mTor pathway is involved. No modulation of mTor activation was observed after siRNA β-catenin.

We also have demonstrated, for the first time, the existence of a positive feedback regulatory loop involving β-catenin and FASN. Specifically, we have shown that inhibition of FASN by using siRNA leads to a significant downregulation of β-catenin expression in MCL cells. In view of the biological significance of β-catenin in MCL [28], our findings suggest that one mechanism by which FASN exerts its oncogenic function in MCL cells is by upregulating β-catenin. These new information suggests that inhibiting FASN and β-catenin in combination may be a useful therapeutic approach.

Despite the fact that FASN overexpression has been observed in different forms of cancer, there is still no clear mechanism explaining how FASN mediates its oncogenic effects. It has been postulated that FASN, by virtue of its normal functions, provide a source of fatty acids for membrane production as well as energy supply [9], [37]. Moreover, FASN has been associated with post-translational modifications (such as palmitoylation) of proteins [29], a phenomenon that has been described to promote the activation of the Src-family tyrosine kinases [38]. As mentioned above, FASN may mediate its oncogenic effects by upregulating β-catenin, a protein known to carry oncogenic functions.

To conclude, the present study describes the high level of FASN expression is a consistent finding in MCL cell lines and tumors. Our data has supported the concept that FASN confers anti-apoptotic effects in MCL cells. Our results also uncovered a positive feedback loop involving FASN and β-catenin, a signaling protein previously reported to be important in the pathobiology of MCL. Thus, inhibition of FASN, possibly in combination with the blockade of β-catenin, may be a useful approach to treat MCL.

## Supporting Information

Figure S1 **Downregulation of β-catenin with the use of two different siRNA sequences (labeled 1 and 2).** Both siRNA species induced a dramatic decrease in FASN protein detectable by western blots. Two MCL cell lines, Jeko-1 (A) and Mino (B), were used for this experiment. Cell lysates were prepared 48 hours after the siRNA transfection.
(TIF)
