# Supplementary figures and images for: Blockade of Fatty Acid Synthase Triggers Significant Apoptosis in Mantle Cell Lymphoma
Source: PLoS One. 2012 Apr 2;7(4):e33738. doi: 10.1371/journal.pone.0033738 (PMC3317445; doi:10.1371/journal.pone.0033738)

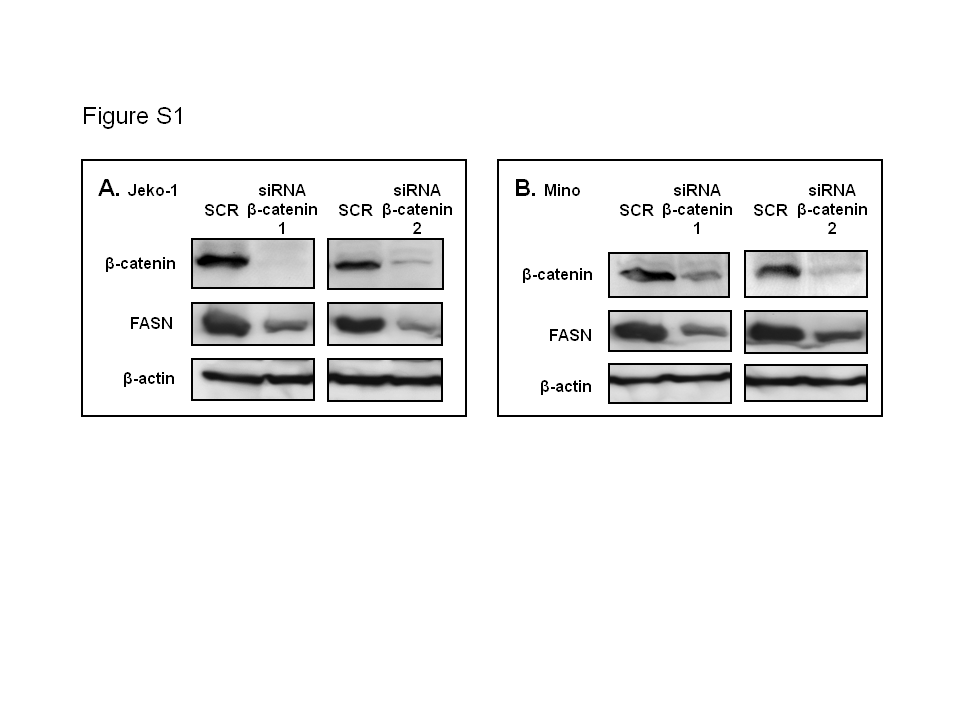

Supplement: Figure S1 — Downregulation of β-catenin with the use of two different siRNA sequences (labeled 1 and 2). Both siRNA species induced a dramatic decrease in FASN protein detectable by western blots. Two MCL cell lines, Jeko-1 (A) and Mino (B), were used for this experiment. Cell lysates were prepared 48 hours after the siRNA transfection. (TIF) [file pone.0033738.s001.tif]
